# Impact of thymoquinone on the Nrf2/HO-1 and MAPK/NF-κB axis in mitigating 5-fluorouracil-induced acute kidney injury *in vivo*

**DOI:** 10.3389/fonc.2025.1572095

**Published:** 2025-05-26

**Authors:** Summya Rashid

**Affiliations:** College of Pharmacy, Prince Sattam Bin Abdulaziz University, Al Kharj, Saudi Arabia

**Keywords:** thymoquinone, p38MAPK, NF-kB, inflammation, oxidative stress, redox signaling, Nrf2/HO-1 signaling pathway

## Abstract

**Background:**

Chemotherapy-induced organ toxicity is one of the most common toxic effects of 5-fluorouracil (5-FU) in cancer patients. Therefore, new strategies are needed to prevent chemotherapy-induced kidney toxicity. Thymoquinone (TQ), a constituent of the plant *Nigella sativa* from the family Renunculaceae, has been found to be antiapoptotic, antioxidant, antimicrobial, anti-inflammatory, and protective against renal damage. This study aims to evaluate the effect of TQ in preventing nephrotoxicity induced by 5-FU treatment.

**Method:**

Male albino Wistar rats were divided into four groups and administered saline (group I), 5-FU (150 mg/kg; group II), 5-FU+TQ (50 mg/kg; group III), and 5-FU+TQ (100 mg/kg; group IV). On the 21st day, rats were killed, and biochemical, histological, serological, and molecular analyses were conducted using kidney tissues and blood samples.

**Results:**

5-FU induced kidney injury, as evidenced by alterations in kidney function markers (BUN, Cr, LDH, KIM-1), lipid peroxidation (LPO), ROS generation, histological changes, and a reduction in antioxidant defense mechanism (GSH, GR, GPx, and CAT). Additionally, 5-FU triggered crosstalk between Nrf2 and NF-κB/p38MAPK axis by significantly upregulating p-p38, p-JNK, p-ERK1/2, p-NF-κB, TNF-α, IL-1β, TGF-β, and IL-6, while downregulating Nrf2 and HO-1, resulting in kidney damage. Pre-, post-, and cotreatment with TQ alleviated kidney injury by replenishing antioxidant reserves, reducing serum toxicity, decreasing ROS generation and lipid peroxidation, downregulating p38 MAPK/NF-κB axis/pathway proteins, and upregulating Nrf2 and HO-1, thereby enhancing antioxidant axis and restoring kidney architecture.

**Conclusion:**

Based on the results obtained in the present study, TQ appears to be a beneficial agent that could be used as an adjuvant therapy for the prevention of 5-FU-induced nephrotoxicity.

## 1 Introduction

5-Fluorouracil (5-FU) is an antimetabolite and antineoplastic agent with notable efficacy against various malignancies, including cancers of the head and neck, breast, skin, stomach, and colorectum (1). It is a pyrimidine analog that, upon metabolism, is converted to 5-fluoro-2-deoxyuridine monophosphate, which inhibits thymidylate synthase. This ultimately suppresses thymine nucleotide production, resulting in the inhibition of cell proliferation, DNA damage, and apoptosis in both cancerous and normally replicating cells, leading to widespread adverse effects (2). Consequently, 5-FU is associated with leucopenia, mucositis, and toxicities affecting the heart, liver, and kidneys, which limit its clinical utility. Furthermore, 5-FU undergoes metabolic cleavage into α-fluoro-β-alanine, ammonia, and urea, which contributes to renal damage—one of the major limitations of its clinical use (3). Reports suggest that reactive oxygen species (ROS) promote the oxidation of lipid membranes, proteins, and cells, leading to oxidative stress and inducing toxic effects such as necrosis and apoptosis. These processes play a critical role in mediating 5-FU-induced kidney damage (4). Various studies indicate that ROS and inflammatory mediators also activate the mitogen-activated protein kinases (MAPK) pathway, which includes p38 MAPK, Jun N-terminal kinase (JNK), and extracellular signal-regulated kinase (ERK). This pathway regulates multiple cellular processes, including stress adaptation, proliferation, differentiation, and apoptosis (5). Moreover, stimulation of the ERK pathway activates transcription factors such as nuclear factor kappa B (NF-κB), which regulates downstream inflammatory mediators/cytokines and protein expressions (6). The MAPK pathway interacts with NF-κB and modulates inflammatory processes in various pathological conditions, making the MAPK/NF-κB signaling pathway a key coordinator of inflammation, as previously reported in 5-FU studies (7). In addition to oxidative stress, 5-FU-induced ROS generation can activate various stress signaling pathways, such as NF-κB, which promotes the expression of inflammatory cytokines and molecules including tumor necrosis factor-α (TNF-α), transforming growth factor-β (TGF-β), interleukin (IL)-1β, and IL-6. These factors play a central role in the pathological features of 5-FU-induced kidney inflammation. NF-κB activation involves the phosphorylation and degradation of IκB by IκK. Under conditions of ROS and inflammation, IκK and IκB activation promote NF-κB phosphorylation, resulting in an irreversible inflammatory response. These proinflammatory mediators initiate a positive feedback loop, amplifying NF-κB activation and worsening proinflammatory signaling, thereby exacerbating kidney damage (8, 9).

Previous studies have shown that oxidant–antioxidant imbalance caused by free radicals and ROS leads to the activation of redox-sensitive signaling pathways such as the nuclear-factor-erythroid-2-related factor 2 (Nrf2)–Kelch ECH-associated protein 1 (Keap1) pathway. These signaling pathways play a critical role in mitigating kidney pathophysiology, as the Nrf2–Keap1 axis is one of the most important cytoprotective mechanisms against oxidative stress. It plays a key role in protecting the kidneys from various pathological conditions (10). Nrf2 is a leucine zipper protein that regulates the expression of downstream protective and antioxidant genes, including NAD (P)H quinone dehydrogenase 1 (NQO-1) and heme oxygenase 1 (HO-1). Under untressed conditions, Nrf2 is rapidly degraded in the cytoplasm by a complex of proteins. Keap1 facilitates Nrf2 ubiquitination by interacting with specific cysteine residues. Under abnormal conditions, such as increased ROS generation, Nrf2 ubiquitination is reduced, allowing it to translocate to the nucleus, where it activates the transcription of downstream genes that protect against oxidative damage and inflammation. Therefore, the Nrf2 signaling pathway is associated with the attenuation of drug-induced ROS production and related organ toxicity/injury (11, 12).

Hence, natural agents or compounds with antioxidant/anti-inflammatory properties, or those that help mitigate redox signaling and associated inflammation, may be suitable candidates to restrain 5-FU-enhanced kidney damage via the Nrf2/Keap1 and p38-MAPK/NF-κB pathways, thus providing striking therapeutic signaling targets for drug discovery. There has been a revival of interest in natural agents of medical importance for the management or treatment of various disease conditions as therapeutic agents, due to their minimal toxicity and cost-effectiveness for the public. Reports show that organ toxicities induced by chemotherapeutic drugs have been managed by agents with antioxidative, anti-inflammatory, and antiapoptotic effects (13, 14). Henceforth, antioxidant and anti-inflammatory agents with minimal adverse effects may be used in adjuvant therapy for the intervention against 5-FU-associated renal injury (15). Thymoquinone (TQ), one of the constituents of the plant *Nigella sativa* from the family Renunculaceae, commonly known as black cumin, is chemically 2-methyl-5-isopropyl-1,4-benzoquinone. It holds medicinal importance in the Indian subcontinent and the Arab world, where it has been traditionally used in Ayurvedic and Unani medicinal systems for the management of various conditions, including hypertension, gastrointestinal problems, respiratory disorders, skin disorders, and obesity (16). Oral *Nigella sativa* extracts, known for their anti-inflammation and antioxidation efficacy, have been used in recent clinical studies to intervene in oxidation and inflammation-driven, unregulated cellular signaling pathways. TQ offers a comprehensive range of valuable biological and pharmacological effects, including antitumor, antioxidant, anti-inflammatory, and protective actions for the kidneys, brain, liver, and heart, as well as immune-modulatory effects (17–19). Consequently, TQ shows regulatory potential in various pathological conditions, including heart disease, arthritis, diabetes mellitus, asthma, atherosclerosis, neurodegenerative disorders, and cancer, which can be attributed to the lipophilic quinine component in its structure. This lipophilic component efficiently and readily enters cellular and subcellular structures, as well as associated transcription factors and kinases, which may be involved in the deregulation of numerous signaling pathways (20–22). Thus, whether TQ can protect against 5-FU-induced kidney injury remains to be explored. Accordingly, the current study aims to provide insight into the molecular mechanisms underlying 5-FU-induced kidney injury, with a specific focus on the role of TQ in regulating redox signaling, MAPKs, NF-κB, and Keap1/Nrf2 pathways.

## 2 Materials and methods

### 2.1 Chemicals

All chemicals, including 5-FU and TQ, were of high grade. Chemicals were purchased from Sigma-Aldrich (USA) and Thermo Fisher Scientific (USA). The following chemicals and reagents were used: catalase (CAT), malondialdehyde (MDA), glutathione peroxidase (GPx), reduced glutathione (GSH), glutathione reductase (GR), ascorbic acid, ferric chloride, trichloroacetic acid (TCA), thiobarbituric acid (TBA), 2′7′-dichlorodihydrofluorescein diacetate, hydrogen peroxide (H_2_O_2_), phenol red, sodium hydroxide (NaOH), sulfosalicylic acid, 5,5′-dithiobis-(2-nitrobenzoic acid) (DTNB), ethylenediaminetetraacetic acid (EDTA), magnesium chloride (MgCl), potassium chloride (KCl), nicotinamide adenine dinucleotide phosphate (NADPH), sodium azide, sodium tungstate, kidney injury marker (KIM-1), sodium pyruvate, NF-kB-p65 rabbit polyclonal antibody, p-p38 MAPK rabbit antibody, biotinylated Goat Anti-Polyvalent Plus, streptavidin peroxidase plus, 3,3′-diaminobenzidine, hematoxylin, DPX, Trizol reagent, and commercial kit (eBioscience, USA).

### 2.2 Male albino Wistar rats and experimental protocol

Animal experiments & handling were directed as guidelines given by animal ethics and permitted by Ethics Committee of Prince Sattam Bin Abdulaziz University, Al Kharj having ethical clearance no. SCBR-492/2025. Wistar rats (170–200 g), male, albino, four- to six week-old. housed in a meticulous environment having standard living conditions like temperature and humidity with discontinuous 12 h light and dark cycle. Rats had food and water access freely. Rats (n = 24) were arbitrarily separated as six rats each in one group and total groups were four. Animals adapted themselves for 1 week in animal facility preceding to the start of experimentation.

Rats in group I were treated with water orally for 20 days. TQ was administered at two doses, 50 and 100 mg/kg b.w., to groups III and IV. On the 20th day, 5-FU (150 mg/kg body weight) was administered intraperitoneally to groups II, III, and IV. Male albino Wistar rats were killed by cervical dislocation under mild anesthesia using a xylazine/ketamine combination, 24 h after 5-FU administration. The treatment regimen given is detailed in **Table 1**. Blood was collected to obtain serum for marker enzyme estimations. Kidneys were excised, cleaned, and stored for further analysis, including biochemical estimations, immunohistochemistry, reverse transcriptase–polymerase chain reaction, ELISA, and histology (15, 22–24).

**Table 1:** Schematic treatment regimen.

Groups (*n* = 6)	Treatment from the first to the 20th day	Treatment on the 19th day
Group I (control)	Water	Normal saline only (i.p.)
Group II (5-FU)	Water	5-FU (150 mg/kg b.w. i.p.; 19th day)
Group III (5-FU+TQ1) (50 mg/kg b.w.)	TQ1 (50 mg/kg b.w.)	5-FU (150 mg/kg b.w. i.p.; 19th day)
Group IV (5-FU + TQ2) (100 mg/kg b.w.)	TQ2 (100 mg/kg b.w.)	5-FU (150 mg/kg b.w. i.p.; 19th day)

### 2.3 Preparation of kidney homogenates

The kidneys were excised, and excess tissue was removed. The kidneys were rinsed with chilled normal saline. A 10% (w/v) tissue homogenate was prepared in 0.1 M Tris hydrochloride using a homogenizer set at 2,500 rpm. The homogenate was centrifuged at 5,000 rpm for 20 min at 4°C using a cooling centrifuge. After centrifugation, the upper clear layer/supernatant was used to analyze several biochemical markers, including CAT, MDA, GPx, reduced GSH, and GR. Optical density (OD)/wavelength was quantified using a UV-1601 spectrometer (Shimadzu, Japan), and an ELISA plate reader was used to estimate antioxidant status, protein expressions, and inflammation (25).

### 2.4 Assessment of lipid peroxidase

Lipid peroxidation (LPO) was determined at pH 7.4 using the following reaction mixture: 0.2 mL of supernatant, 0.2 mL of 100 mM ascorbic acid, 0.58 mL of 0.1 M phosphate buffer (PB), and 0.02 mL of 100 mM ferric chloride. The mixture was incubated at 37°C for 60 min in a shaking water bath. Afterward, 1,000 µL of 10% trichloroacetic acid and 10 mL of 0.67% thiobarbituric acid were added. The reaction tubes were then transferred to an ice bath. After centrifugation for 10 min at 2,500×*g*, the OD was measured at 532 nm (26).

### 2.5 Assessment of ROS

ROS levels were assessed. Upon oxidation, 2′7′-dichlorodihydrofluorescein diacetate is converted to 2′7′-dichlorofluorescein, which is used to measure reactive oxygen species, with OD measured at 430 nm (27).

### 2.6 Assessment of hydrogen peroxide

H_2_O_2_ levels were evaluated. Microsomes were prepared according to the method described by Goldstone et al. Normal rat kidney microsomes were isolated from kidney homogenates in isotonic sucrose containing 0.05 M Tris-HCl (pH 7.5), 0.005 M MgCl, and 0.025 M KCl (6). The crude homogenate was centrifuged at 10,000×*g* for 10 min, and the postmitochondrial supernatant was carefully decanted. Microsomes were obtained by centrifugation at 105,000×*g* for 1 h. A 2,000-µL volume of the microsomes was obtained with phenol red in a 1,000-µL reaction mixture and incubated at 37°C for –1 h. To stop the reaction, 10 µL of 10 N NaOH was added, and the mixture was centrifuged at 800×*g* for 5 min (28).

### 2.7 Assessment of CAT activity

CAT was determined by mixing 50 mL of 10% postmitochondrial supernatant, 1,950 mL of 0.1 M phosphate buffer, and 1,000 mL of 0.10 mM H_2_O_2_ at physiological pH. The absorbance was measured at 405 nm (29).

### 2.8 Estimation of antioxidant enzyme armory

#### 2.8.1 Estimation of GSH

GSH was assessed as previously described. The reaction mixture was prepared by mixing postmitochondrial supernatant (PMS) with 4% sulfosalicylic acid in a 1:1 (v/v) ratio, followed by incubation for 60 min and centrifugation at 1,200×*g* for 15 min at 4°C. Next, 400 mL of the filtered aliquots was mixed with 400 mL of 10 mM DTNB and 2,200 mL of 0.1 M PB (pH 7.4). The OD was measured at 415 nm (30).

#### 2.8.2 Assessment of GR

The experiment was initiated in a final volume of 1 mL by combining 825 µL of 0.1 M phosphate buffer (pH 7.4), 50 mL of 0.5 mM EDTA, 50 mL of 0.1 mM NADPH, 25 mL of 1.0 mM oxidized form of glutathione, and 50 mL of 10% PMS (31). OD was measured at 340 nm.

#### 2.8.3 Estimation of GPx

GPx was determined by combining 100 µL of 1 mM EDTA, 1.44 mL of 0.1 M PB, and 0.1 mL of 1 mM sodium azide at physiological pH. OD was measured at 340 nm (32).

### 2.9 Serum diagnostic renal toxicity marker estimation

At the time of killing, animals were anesthetized, and blood was collected. The blood was centrifuged at 10,000×*g* for 10 min to obtain serum, which was then used to evaluate kidney injury markers.

#### 2.9.1 Assessment of blood urea nitrogen

Blood urea nitrogen (BUN) was assessed following the method by Kanter (1975). An equal volume of 10% TCA was mixed with serum and centrifuged at 2,000 rpm to obtain a protein-free supernatant. To 0.5 mL of this supernatant, 3.5 mL of distilled H_2_O, 0.8 mL of 2% diacetylmonoxime, and 3.2 mL of H_2_SO_4_–H_3_PO_4_ reagents were added, and the mixture was incubated in a boiling water bath for half an hour. After cooling, absorbance was measured at 450 nm (33).

#### 2.9.2 Assessment of creatinine

Creatinine (Cr) was measured by combining 1.0 mL of 5% sodium tungstate, 0.6 M H_2_SO_4_, and distilled H_2_O water with 1.0 mL of serum, followed by centrifugation at 800×*g* for 5 min. The resulting supernatant was then mixed with 1 mL of 1.05% picric acid and 0.75 M NaOH. After 20 min, absorbance was measured at 520 nm (34).

#### 2.9.3 Measurement of kidney injury marker

KIM-1 was measured using a kit from Adipo Bioscience^®^ Inc. (USA), following the manufacturer’s protocol.

#### 2.9.4 Estimate lactate dehydrogenase activity

LDH assessment was performed in serum using a reaction mixture containing 100 µL of NADH (0.02 M), 0.2 mL of serum, 0.1 mL of sodium pyruvate (0.01 M), and 1.1 mL of phosphate buffer (0.1 M) at physiological pH. Absorbance was then measured using a spectrophotometer at 340 nm (35).

### 2.10 Assessment of inflammatory markers and p-p38 MAPK pathway proteins

TNF-α, IL-6, IL-1β, TGF-β, p-p38MAPK, p-ERK1/2, and p-JNK were measured using a commercial kit (eBioscience, USA). The complete procedures were carried out according to the manufacturer’s protocol through ELISA.

### 2.11 Immunohistochemistry of NF-κB and p-p38 MAPK

Immunohistochemistry (IHC) was performed to recognize NF-κB and p-p38 MAPK protein expressions in kidney tissue. The IHC staining procedure was completely followed as explained by Rashid et al. (25). NF-kB-p65 rabbit polyclonal antibody (1:150), and p-p38 MAPK rabbit antibody (1:150) (Thermo Fisher Scientific, USA) were used and incubated overnight at 4°C. The following day, slides were washed three times in Tris buffer (pH 6.0) and incubated with biotinylated Goat Anti-Polyvalent Plus (Thermo Fisher Scientific, USA) at room temperature for 30 min. Washing with Tris buffer was further done, and slides were incubated with streptavidin peroxidase plus (Thermo Fisher Scientific, USA) at room temperature. After additional washing with Tris buffer, 3,3′-diaminobenzidine was used to develop the immunostaining reaction product. Following this, slides were washed with distilled water, and counterstaining was performed using hematoxylin before leaving the slides to dry. DPX was used to mount the sections, which were then protected with coverslips. The slides were prepared for viewing.

### 2.12 Gene expression studies via RT-PCR

RNA isolation was performed from frozen kidney samples of all groups using Trizol reagent. RNA concentration was measured using Nanodrop, with samples having an A260/A280 nm ratio > 1.7, and then reverse transcribed into cDNA. SYBR Green master mix was used to amplify cDNA along with primers. Data were analyzed using the 2−ΔΔCt method (36) and normalized to GAPDH as the internal control. Amplification was performed for Nrf2 [5′-TTGTAGATGACCATGAGTCGC C-3′ (sense), 5′TGTCCTGCTGTATGCTGCTT-3′ (antisense)], HO-1 [5′GTAAATGCAGTGTTGGCCCC-3′ sense), 5′-ATGTGCCAGGCATCTCCTTC-3′ (antisense)], and IL-1ß [5′-AATACCACTTGTTGGCTTA-3′ (sense), 5′TGTGATGTTCCCATTAGAC-3′ (antisense)] (24).

### 2.13 Calculation of the protein

Protein concentration was measured using the Lowry method, with the OD assessed at 280 nm (37).

### 2.14 Histology

The kidneys were excised, cleaned in PB, and fixed in 10% neutral buffered formalin. The tissues were processed automatically, and kidney specimens were embedded in paraffin. Sections of 4-µm thickness were cut using a microtome and stained with hematoxylin and eosin on glass slides.

### 2.15 Statistical analysis

Data are presented as mean ± standard error of the mean (SEM) for each group. Variances between sets/groups were determined using analysis of variance (ANOVA), followed by the Tukey–Kramer multiple comparisons test. Statistical significance is indicated by *p* < 0.05 unless otherwise noted.

## 3 Results

### 3.1 TQ diminishes ROS and H_2_O_2_ levels

**Figure 1:**
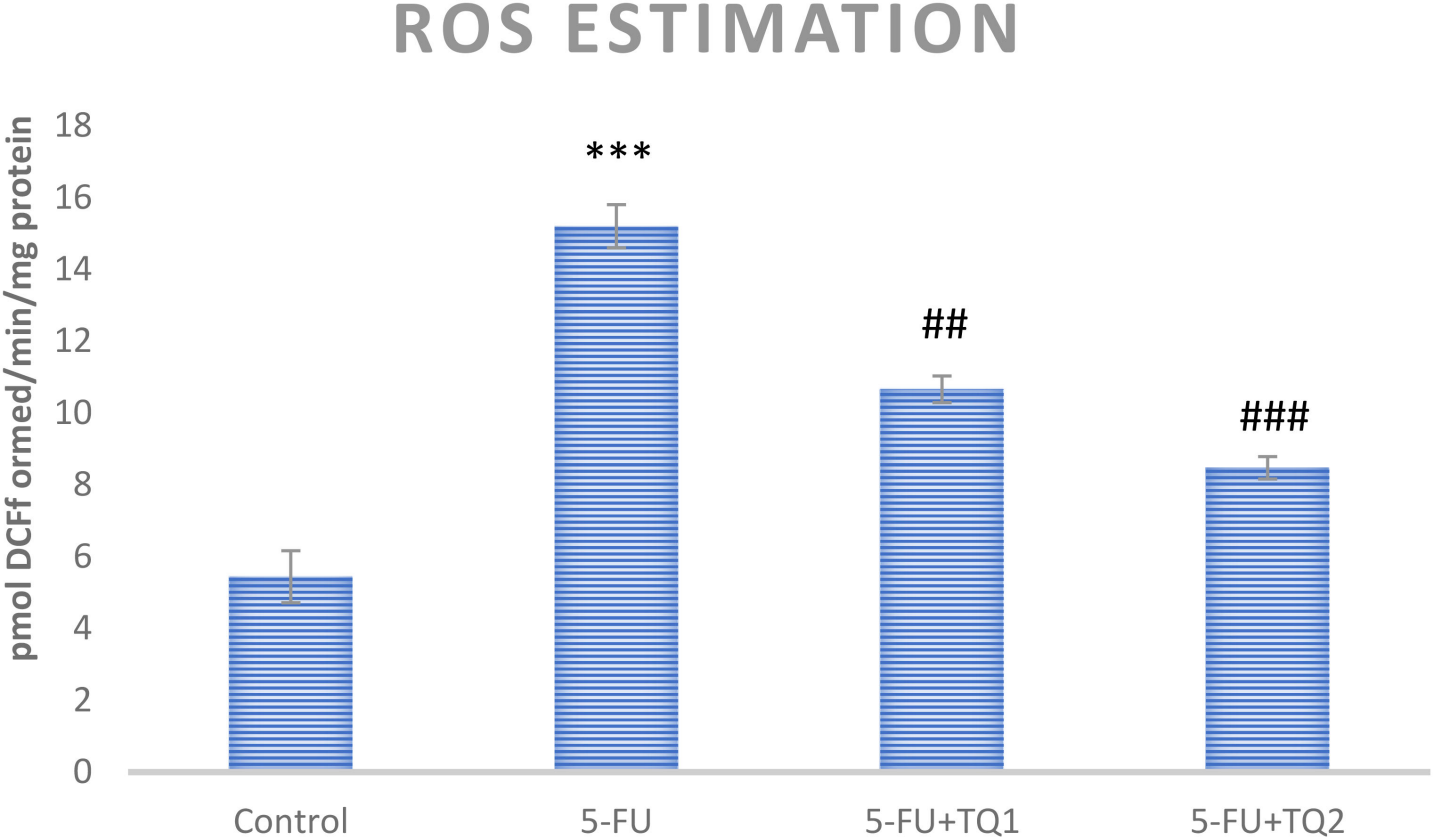
Effect of low and high prophylactic doses of thymoquinone (TQ) on ROS levels in 5-fluorouracil (5-FU)-induced kidney damage. A comparison is shown between the 5-FU-treated group and the control group, with statistical significance indicated as ^***^ *p* < 0.001, ^**^ *p* < 0.01, and ^*^ *p* < 0.05. Treatment groups were compared with the 5-FU-treated group: ^#^ *p* < 0.05, ^##^ *p* < 0.01, and ^###^ *p* < 0.001. Group I: normal control, group II: 5-FU-treated (150 mg/kg b.w.), group III: 5-FU-treated (150 mg/kg b.w.) + TQ (lower dose) (50 mg/kg b.w.), and group IV: 5-FU-treated (150 mg/kg b.w.) + TQ (100 mg/kg b.w.).

**Figure 2:**
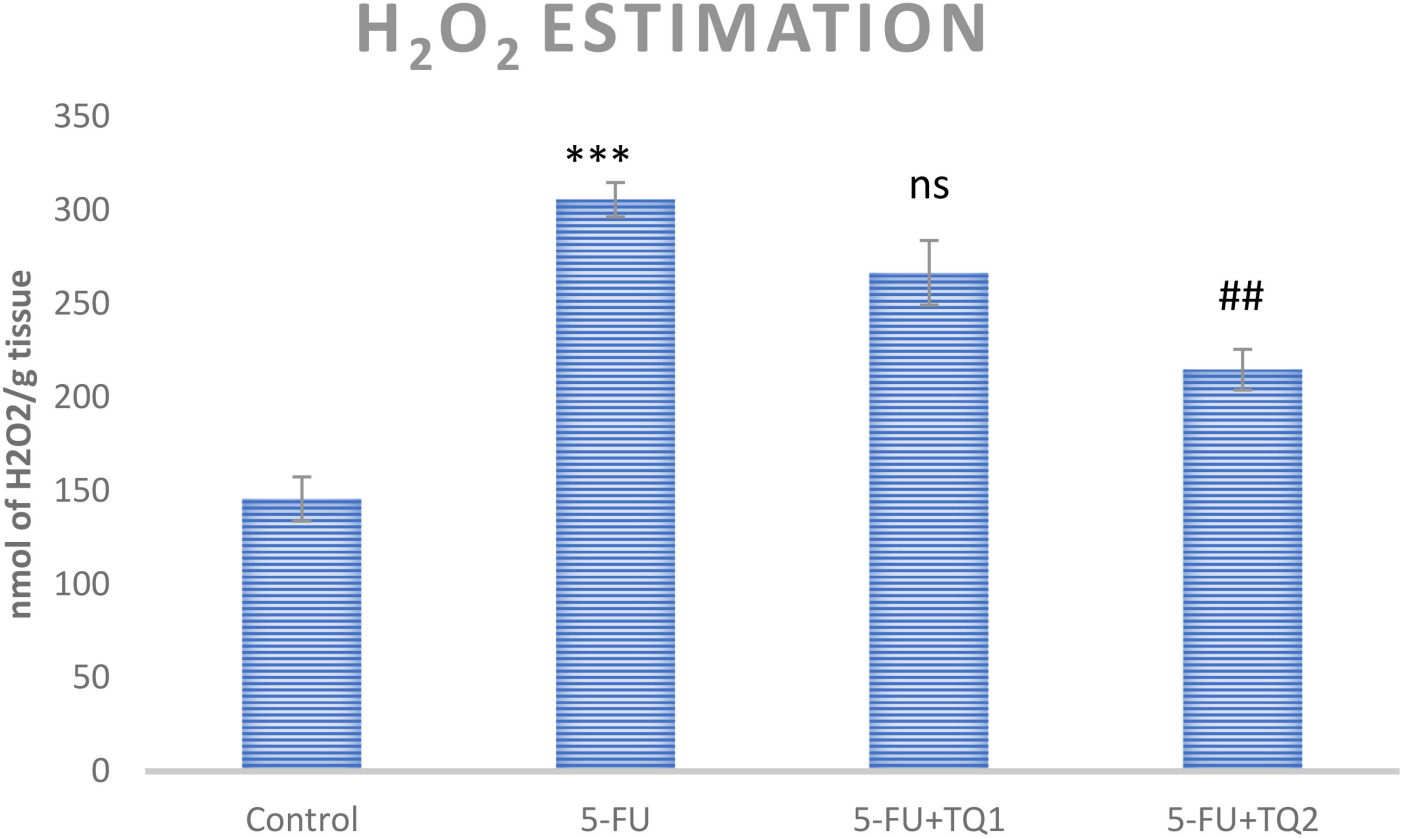
Effect of low and high prophylactic doses of thymoquinone (TQ) on H_2_O_2_ levels in 5-fluorouracil (5-FU)-induced kidney damage. A comparison is shown between the 5-FU-treated group and the control group: ^***^ *p* < 0.001, ^**^ *p* < 0.01, and ^*^ *p* < 0.05. Treatment groups were compared with the 5-FU-treated group: ^#^ *p* < 0.05, ^##^ *p* < 0.01, and ^###^ *p* < 0.001. Group I: normal control, group II: 5-FU-treated (150 mg/kg b.w.), group III: 5-FU-treated (150 mg/kg b.w) + TQ (lower dose) (50 mg/kg b.w.), and group IV: 5-FU-treated (150 mg/kg b.w.) + TQ (100 mg/kg b.w.).

5-FU treatment elevated ROS and H_2_O_2_ levels in group II (*p* < 0.001) compared to group I, indicating increased oxidative stress in the kidneys. However, high-dose TQ alleviated ROS and H_2_O_2_ levels in groups III and IV (*p* < 0.05, *p* < 0.01) (**Figures 1**, **2**).

### 3.2 TQ diminishes MDA levels

There was an increase in MDA levels in group II after 5-FU administration compared to group I (*p* < 0.001).TQ treatment at both doses led to significant renewal (*p* < 0.01, *p* < 0.001) of membrane structure and integrity in renal tissue compared to the 5-FU group (**Table 2**).

**Table 2:** Effect of low and high prophylactic doses of thymoquinone (TQ) on antioxidant and oxidative stress marker in fluorouracil (5-FU)-induced kidney damage.

Groups	MDA (nmol MDA formed/g tissue)	CAT (nmol H_2_O_2_ consumed/min/mg protein)
Control	13.2 ± 0.6	48.10 ± 3.1
5-FU	39.8 ± 3.0^***^	24.21 ± 3.5^***^
5-FU+TQ1	23.2 ± 2.3^##^	36.76 ± 1.5^#^
5-FU+TQ2	17.7 ± 0.4^###^	42.31 ± 1.1^###^

A comparison is shown between the 5-FU-treated group and the control group, with statistical significance indicated as ^***^ *p* < 0.001, ^**^ *p* < 0.01, and ^*^ *p* < 0.05. Treatment groups were compared with the 5-FU-treated group: ^#^ *p* < 0.05, ^##^ *p* < 0.01, and ^###^ *p* < 0.001. Group I: normal control, group II: 5-FU-treated (150 mg/kg b.w.), group III: 5-FU-treated (150 mg/kg b.w.) + TQ (lower dose) (50 mg/kg b.w.), and group IV: 5-FU-treated (150 mg/kg b.w.) + TQ (100 mg/kg b.w.).

### 3.3 TQ restores antioxidant machinery

Administration of 5-FU evidently exhausted kidney GSH reserves and repressed the activities of GPx, GR, and CAT compared to group 1 (*p* < 0.001, *p* < 0.01) (**Tables 2**, **3**). Nonetheless, TQ administration at both doses led to a dose-dependent (TQ-nonsignificant, *p* < 0.05, *p* < 0.01, *p* < 0.001) restoration of GSH reserves and the abovementioned antioxidant enzymes, showing significant improvement compared to the 5-FU group (**Tables 2**, **3**).

**Table 3:** Effect of low and high prophylactic doses of thymoquinone (TQ) on antioxidant reservoirs in fluorouracil (5-FU)-induced kidney damage.

Groups	GSH (nmol GSH/g tissue)	GPx (nmol NADPH oxidized/mg protein)	GR (nmol NADPH oxidized/mg protein)
Control	0.95 ± 0.03	245.1 ± 21.6	211.11 ± 15.5
5-FU	0.37 ± 0.02^***^	110.8 ± 16.8^**^	106.21 ± 12.2^**^
5-FU+TQ1	0.52 ± 0.03^#^	178.55 ± 16.2^#^	162.10 ± 17.1^ns^
5-FU+TQ2	0.81 ± 0.04^###^	196.7 ± 15.2^#^	180.11 ± 17.6^#^

A comparison is shown between the 5-FU-treated group and the control group, with statistical significance indicated as ^***^ *p* < 0.001, ^**^ *p* < 0.01, and ^*^ *p* < 0.05. Treatment groups were compared with the 5-FU-treated group: ^#^ *p* < 0.05, ^##^ *p* < 0.01, and ^###^ *p* < 0.001. Group I: normal control, group II: 5-FU-treated (150 mg/kg b.w.), group III: 5-FU-treated (150 mg/kg b.w.) + TQ (lower dose) (50 mg/kg b.w.), and group IV: 5-FU-treated (150 mg/kg b.w.) + TQ (100 mg/kg b.w.).

### 3.4 TQ protects kidney function markers

**Figure 3:**
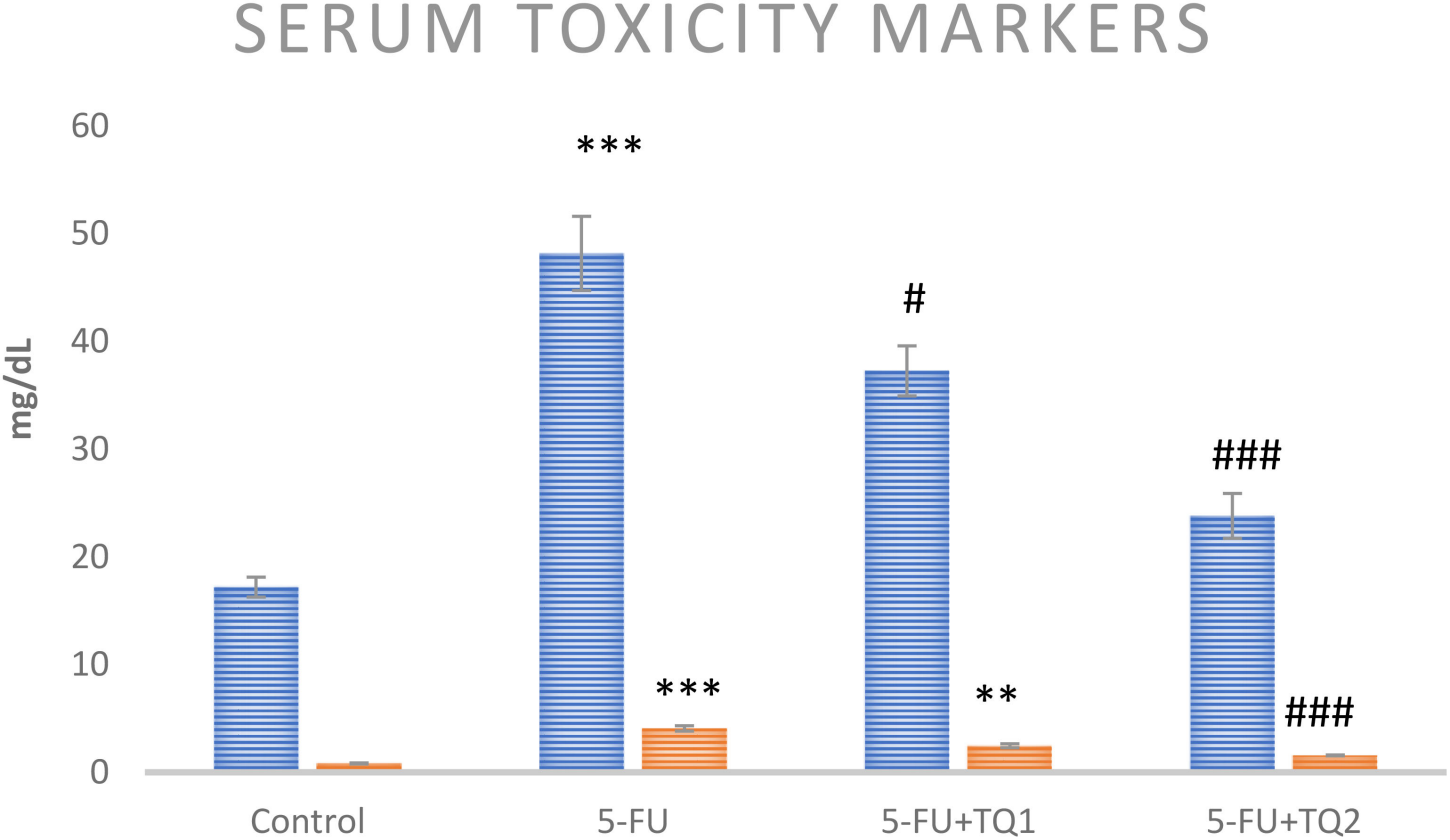
Effect of low and high prophylactic doses of thymoquinone (TQ) on diagnostic serum toxicity markers—BUN (blue) and Cr (orange)—in 5-fluorouracil (5-FU)-induced kidney damage. A comparison is shown between the 5-FU-treated group and the control group ^***^ *p* < 0.001, ^**^ *p* < 0.01, and ^*^ *p* < 0.05. Treatment groups were compared with the 5-FU-treated group: ^#^ *p* < 0.05, ^##^ *p* < 0.01, and ^###^ *p* < 0.001. Group I: normal control, group II: 5-FU-treated (150 mg/kg b.w.), group III: 5-FU-treated (150 mg/kg b.w.) + TQ (lower dose) (50 mg/kg b.w.), and group IV: 5-FU-treated (150 mg/kg b.w.) + TQ (100 mg/kg b.w.).

**Figure 4:**
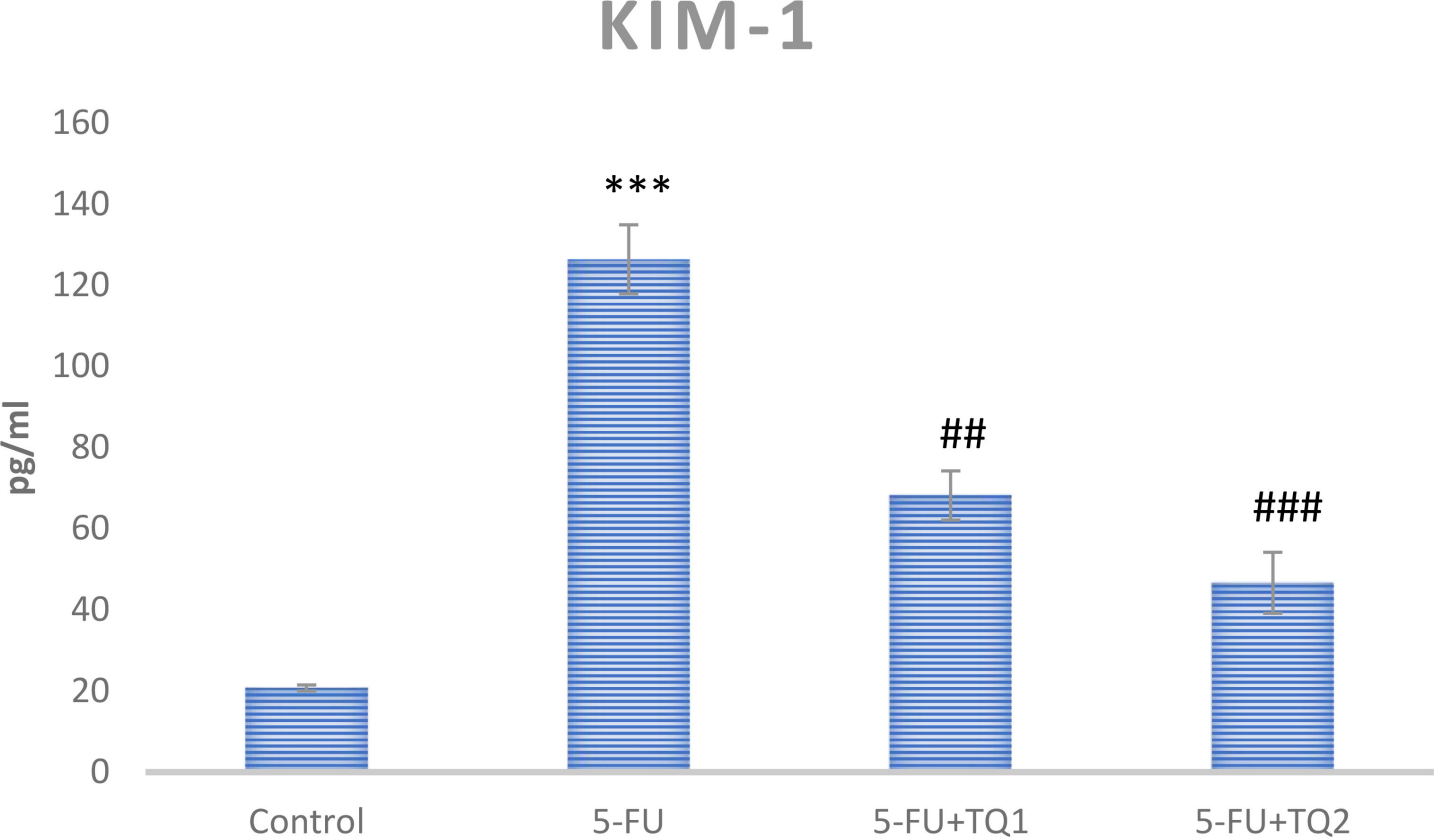
Effect of low and high prophylactic doses of thymoquinone (TQ) on KIM-1 in 5-fluorouracil (5-FU)-induced kidney damage. A comparison is shown between the 5-FU-treated group: ^***^ *p* < 0.001, ^*^ *p* < 0.01, and ^*^ *p* < 0.05. Treatment groups were compared with the 5-FU-treated group: ^#^ *p* < 0.05, ^##^ *p* < 0.01, and ^###^ *p* < 0.001. Group I: normal control, group II: 5-FU-treated (150 mg/kg b.w.), group III: 5-FU-treated (150 mg/kg b.w.) + TQ (lower dose) (50 mg/kg b.w.), and group IV: 5-FU-treated (150 mg/kg b.w.) + TQ (100 mg/kg b.w.).

**Figure 5:**
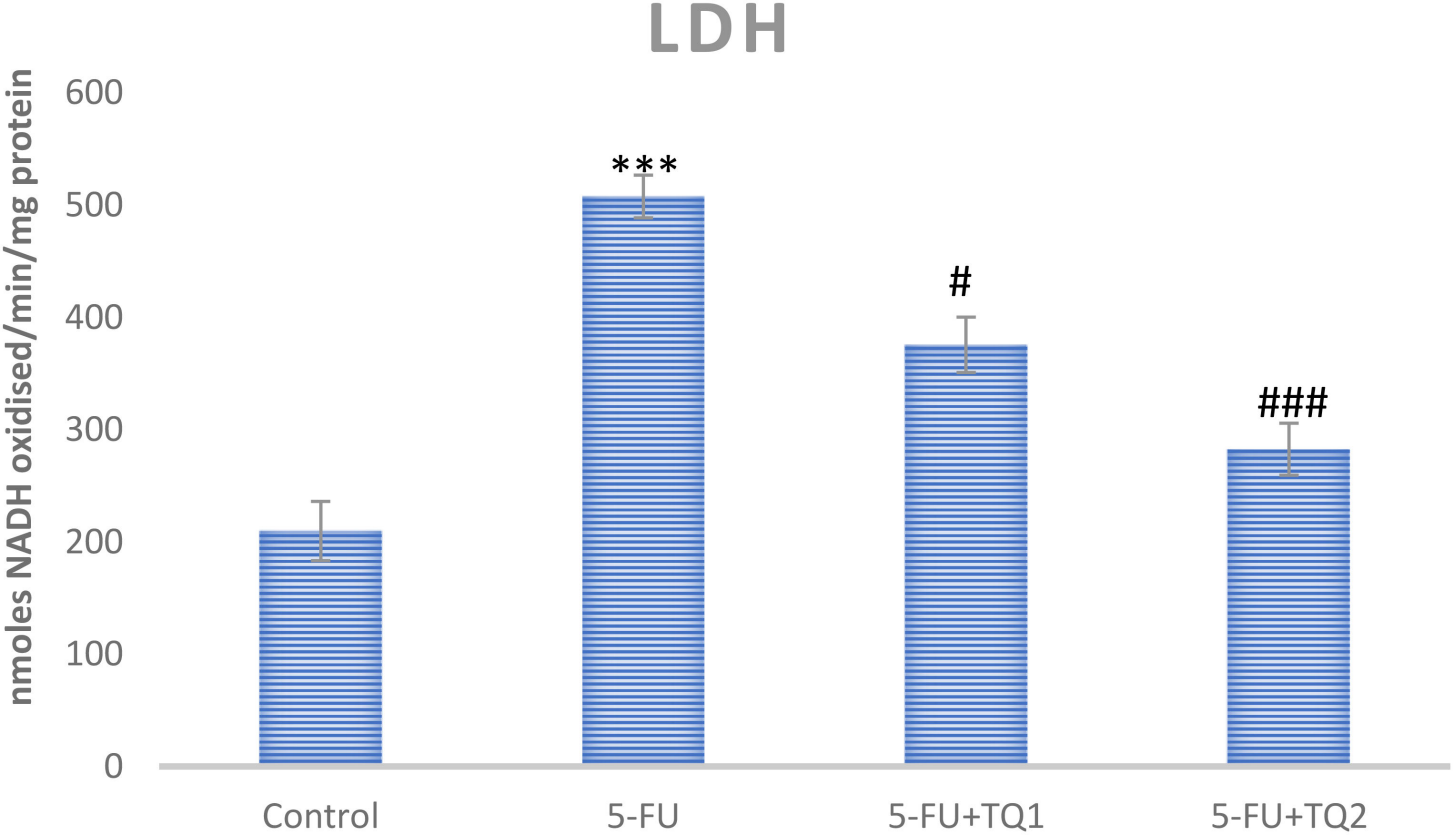
Effect of low and high prophylactic doses of thymoquinone (TQ) on LDH in 5-fluorouracil (5-FU)-induced kidney damage. A comparison between the 5-FU-treated group and the control group: ^***^ *p* < 0.001, ^**^ *p* < 0.01, and ^*^ *p* < 0.05. Treatment groups were compared with the 5-FU-treated group: ^#^ *p* < 0.05, ^##^ *p* < 0.01, and ^###^ *p* < 0.001. Group I: normal control, group II: 5-FU-treated (150 mg/kg b.w.), group III: 5-FU-treated (150 mg/kg b.w.) + TQ (lower dose) (50 mg/kg b.w.), and group IV: 5-FU-treated (150 mg/kg b.w.) + TQ (100 mg/kg b.w.).

Administration of 5-FU demonstrated an elevation in kidney damage/diagnostic biomarkers (BUN, Cr, LDH, and KIM-1) in 5-FU-administered rats in contrast with group I (**Figures 1**, **2**). Group II exhibited raised BUN, Cr, LDH, and KIM-1 (*p* < 0.001) in contrast with group 1 significantly. A noticeable inhibition was observed in BUN, Cr, LDH, and KIM-1 at both doses, respectively, with TQ treatment (*p* < 0.05, *p <*0.01, *p <*0.001) (**Figures 3**–**5**).

### 3.5 TQ treatment regulates inflammatory mediators and p-p38 MAPK kinase pathway proteins

**Figure 6:**
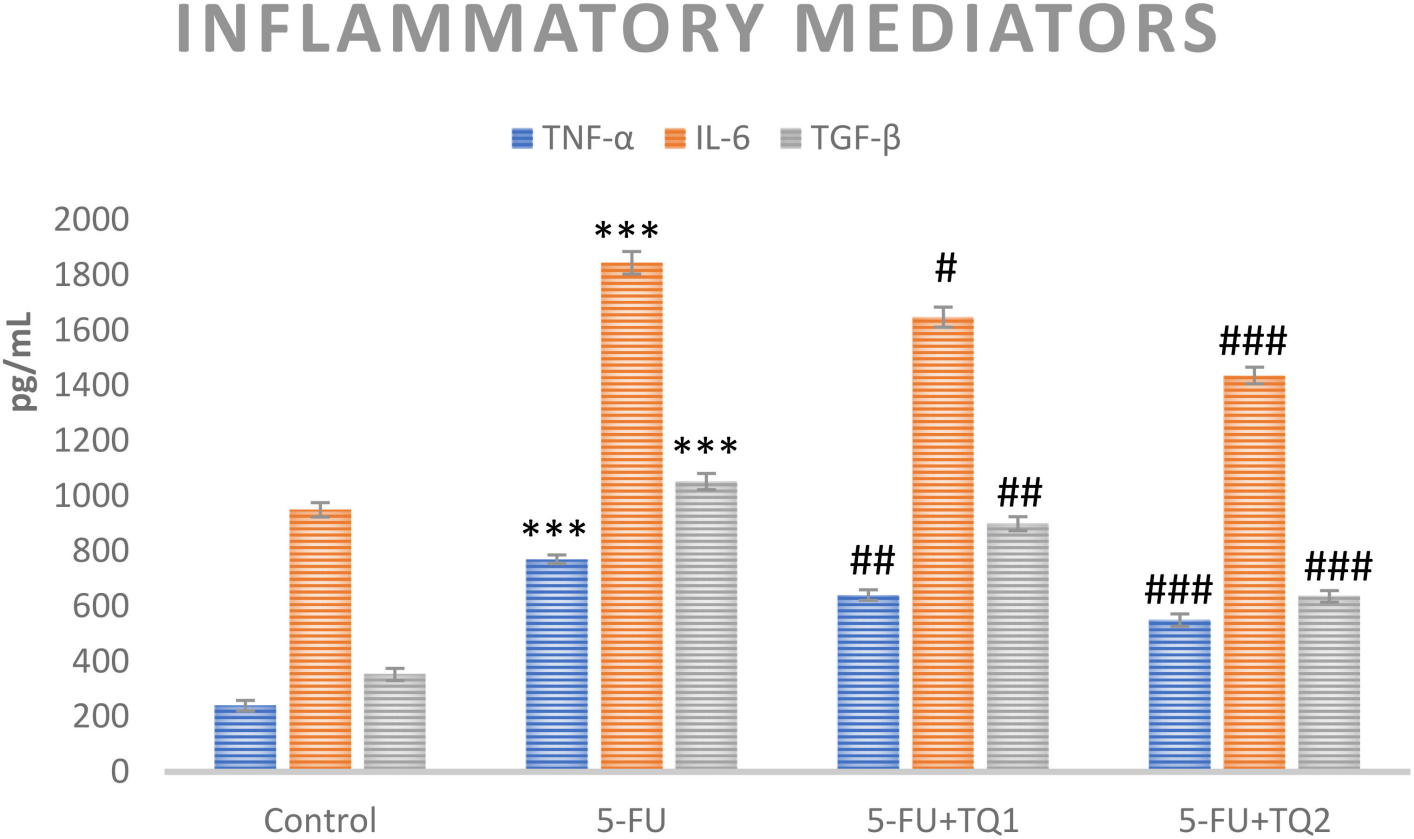
Effect of low and high prophylactic doses of thymoquinone (TQ) on inflammatory mediators in 5-fluorouracil (5-FU)-induced kidney damage. A comparison between the 5-FU-treated group and the control group: ^***^ *p* < 0.001, ^**^ *p* < 0.01, and ^*^ *p* < 0.05. Treatment groups were compared with the 5-FU-treated group: ^#^ *p* < 0.05, ^##^ *p* < 0.01, and ^###^ *p* < 0.001. Group I: normal control, group II: 5-FU-treated (150 mg/kg b.w.), group III: 5-FU-treated (150 mg/kg b.w.) + TQ (lower dose) (50 mg/kg b.w.), and group IV: 5-FU-treated (150 mg/kg b.w.) + TQ (100 mg/kg b.w.).

**Figure 7:**
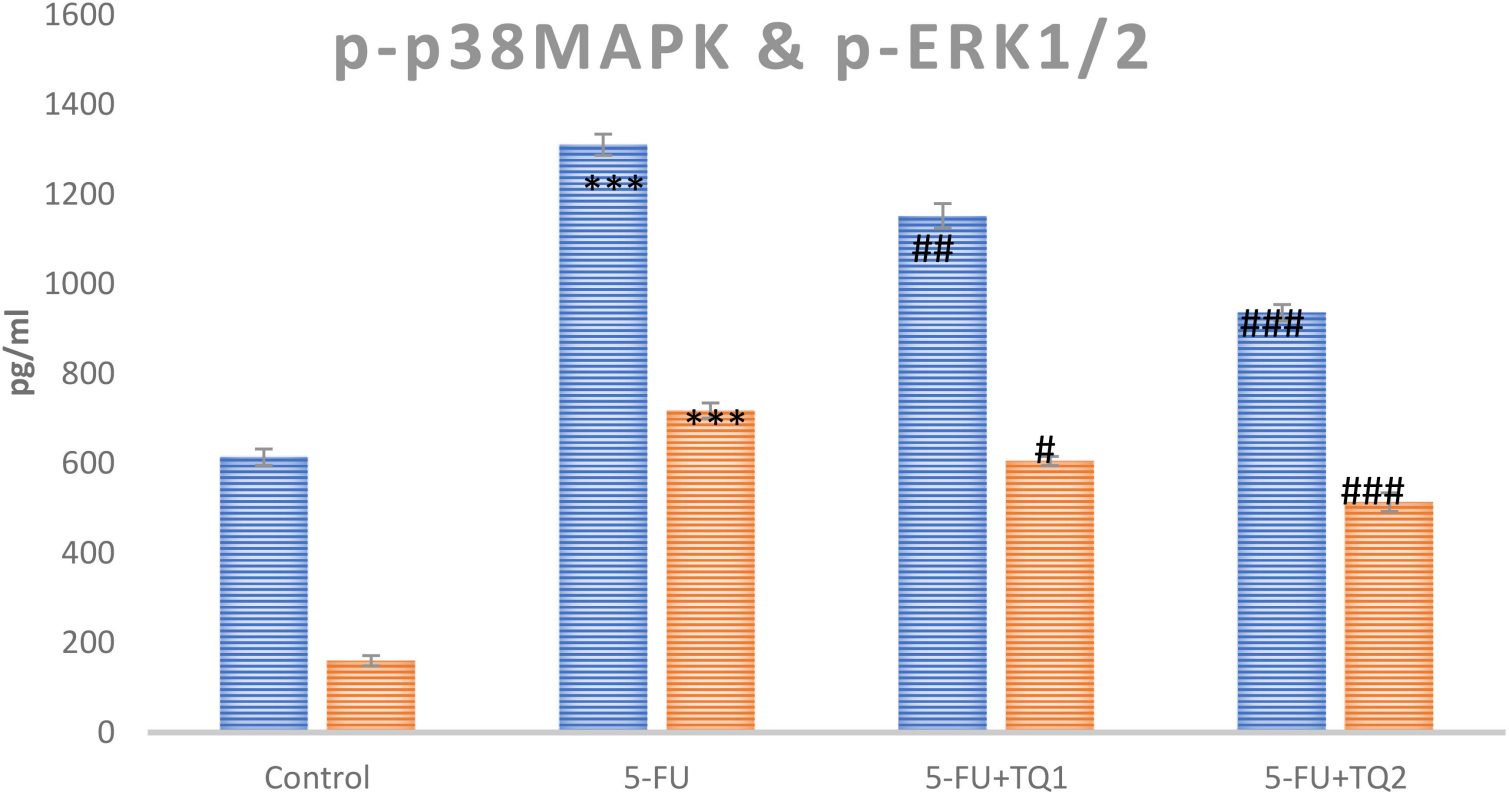
Effect of low and high prophylactic doses of thymoquinone (TQ) on p-p38MAPK protein and pERK1/2 in 5-fluorouracil (5-FU)-induced kidney damage. A comparison between the 5-FU-treated group and the control group: ^***^ *p* < 0.001, ^**^ *p* < 0.01, and ^*^ *p* < 0.05. Treatment groups were compared with the 5-FU-treated group: ^#^ *p* < 0.05, ^##^ *p* < 0.01, and ^###^ *p* < 0.001. Group I: normal control, group II: 5-FU-treated (150 mg/kg b.w.), group III: 5-FU-treated (150 mg/kg b.w.) + TQ (lower dose) (50 mg/kg b.w.), and group IV: 5-FU-treated (150 mg/kg b.w.) + TQ (100 mg/kg b.w.).

Cytokines and p-p38 MAPK kinase pathway proteins, which include TNF-α, TGF-β, IL-6, p-ERK1/2, and p-JNK, respectively, are critical in the development of 5-FU-induced kidney toxicity. 5-FU administration caused a significant upsurge in TNF-α, TGF-β, IL-6, pERK1/2, and pJNK in the 5-FU-administered group in contrast to the control group (**Figures 6**, **7**). Treatment with TQ at both doses markedly downregulated TNF-α, TGF-β, IL-6, p-ERK1/2, and p-JNK, respectively, in contrast to the 5-FU group (*p* < 0.05, *p* < 0.01, *p* < 0.001) (**Figures 3**–**7**), regulating signaling.

### 3.6 TQ promotes Nrf2 signaling

**Figure 8:**
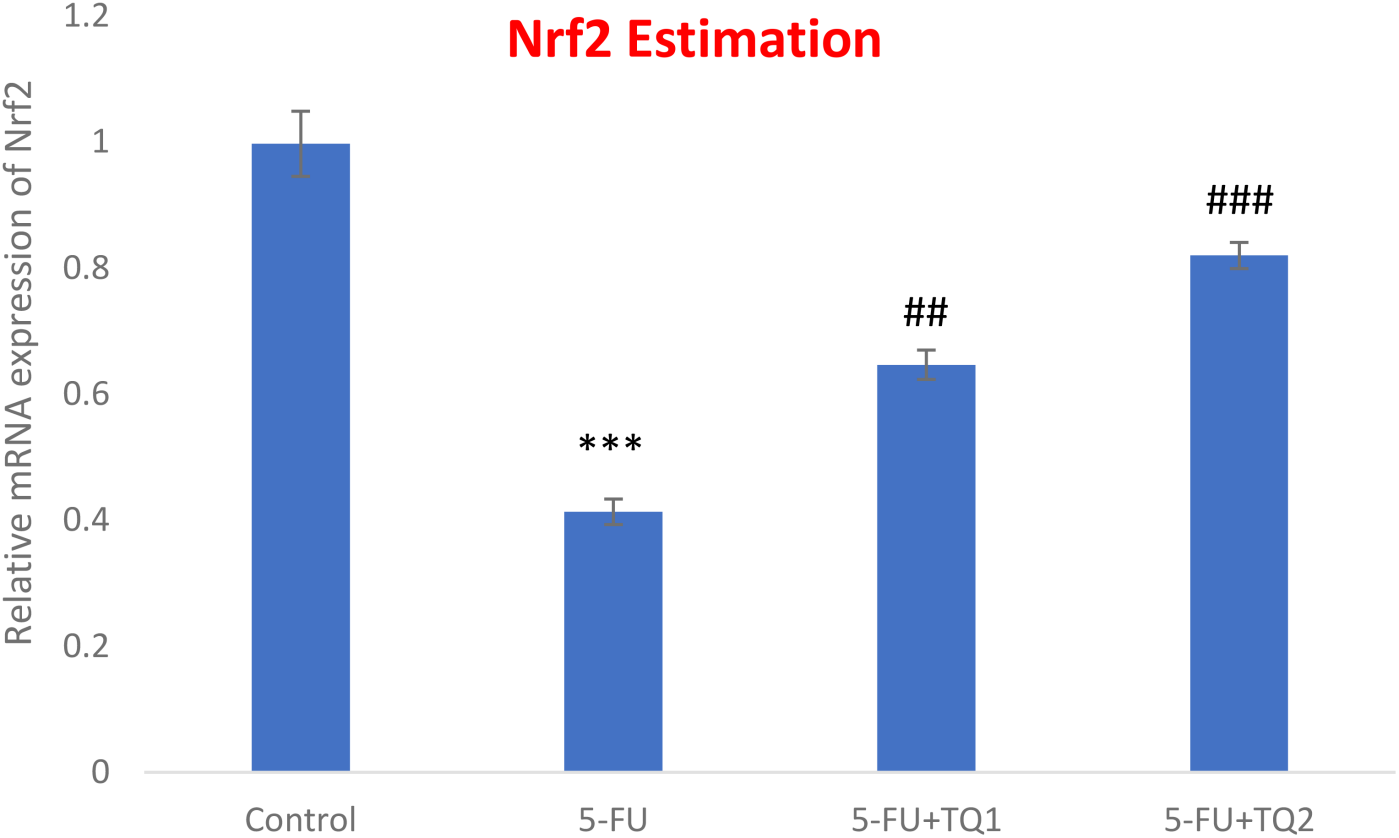
Effect of low and high prophylactic doses of thymoquinone (TQ) on p38 Nrf2 in 5-fluorouracil (5-FU)-induced kidney damage. A comparison between the 5-FU-treated group and the control group: ^***^ *p* < 0.001, ^**^ *p* < 0.01, and ^*^ *p* < 0.05. Treatment groups were compared with the 5-FU-treated group: ^#^ *p* < 0.05, ^##^ *p* < 0.01, and ^###^ *p* < 0.001. Group I: normal control, group II: 5-FU-treated (150 mg/kg b.w.), group III: 5-FU-treated (150 mg/kg b.w.) + TQ (lower dose) (50 mg/kg b.w.), and group IV: 5-FU-treated (150 mg/kg b.w.) + TQ (100 mg/kg b.w.).

**Figure 9:**
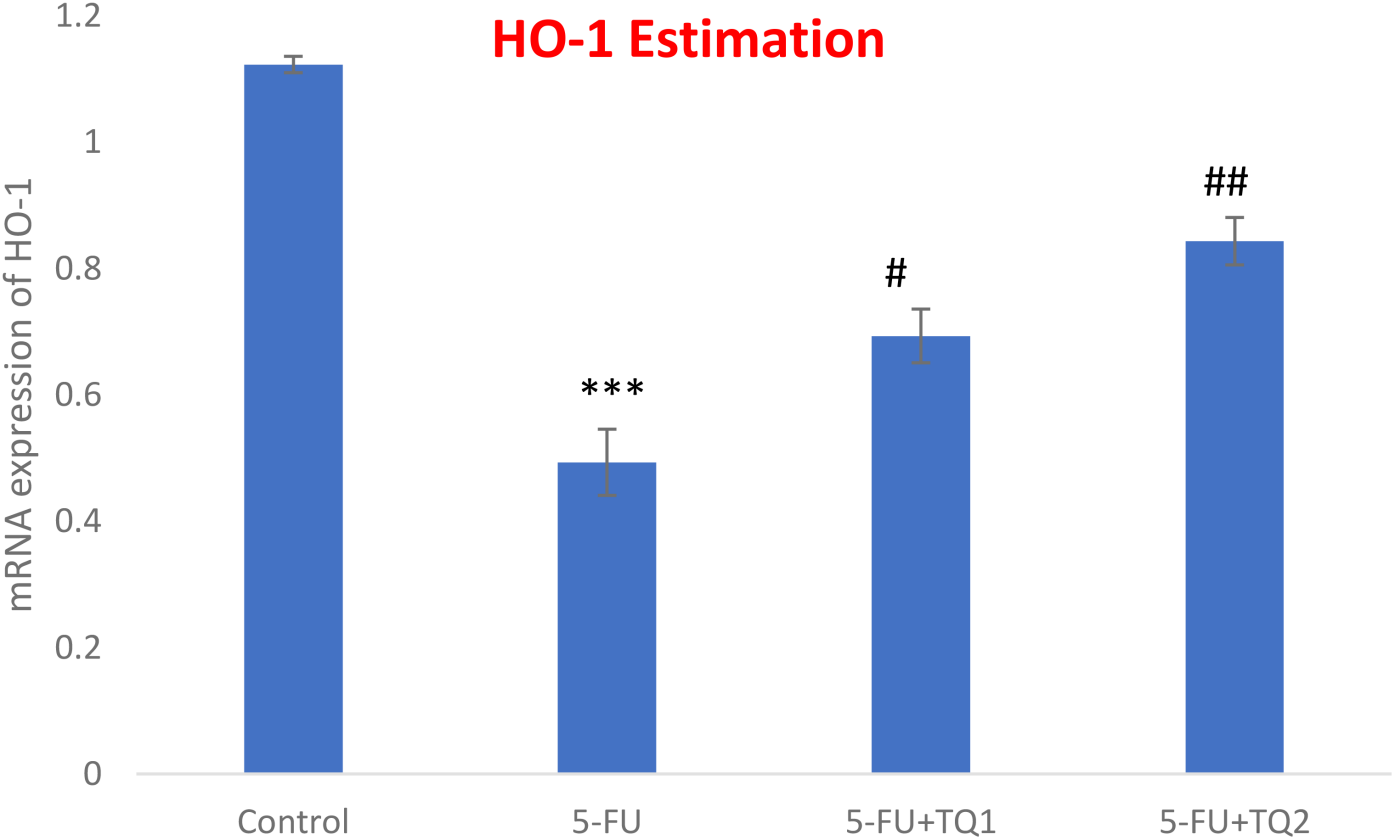
Effect of low and high prophylactic doses of thymoquinone (TQ) on p38 HO-1 in 5-fluorouracil (5-FU)-induced kidney damage. A comparison between the 5-FU-treated group and the control group: ^***^ *p* < 0.001, ^**^ *p* < 0.01, and ^*^ *p* < 0.05. Treatment groups were compared with the 5-FU-treated group: ^#^ *p* < 0.05, ^##^ *p* < 0.01, and ^###^ *p* < 0.001. Group I: normal control, group II: 5-FU-treated (150 mg/kg b.w.), group III: 5-FU-treated (150 mg/kg b.w.) + TQ (lower dose) (50 mg/kg b.w.), and group IV: 5-FU-treated (150 mg/kg b.w.) + TQ (100 mg/kg b.w.).

**Figure 10:**
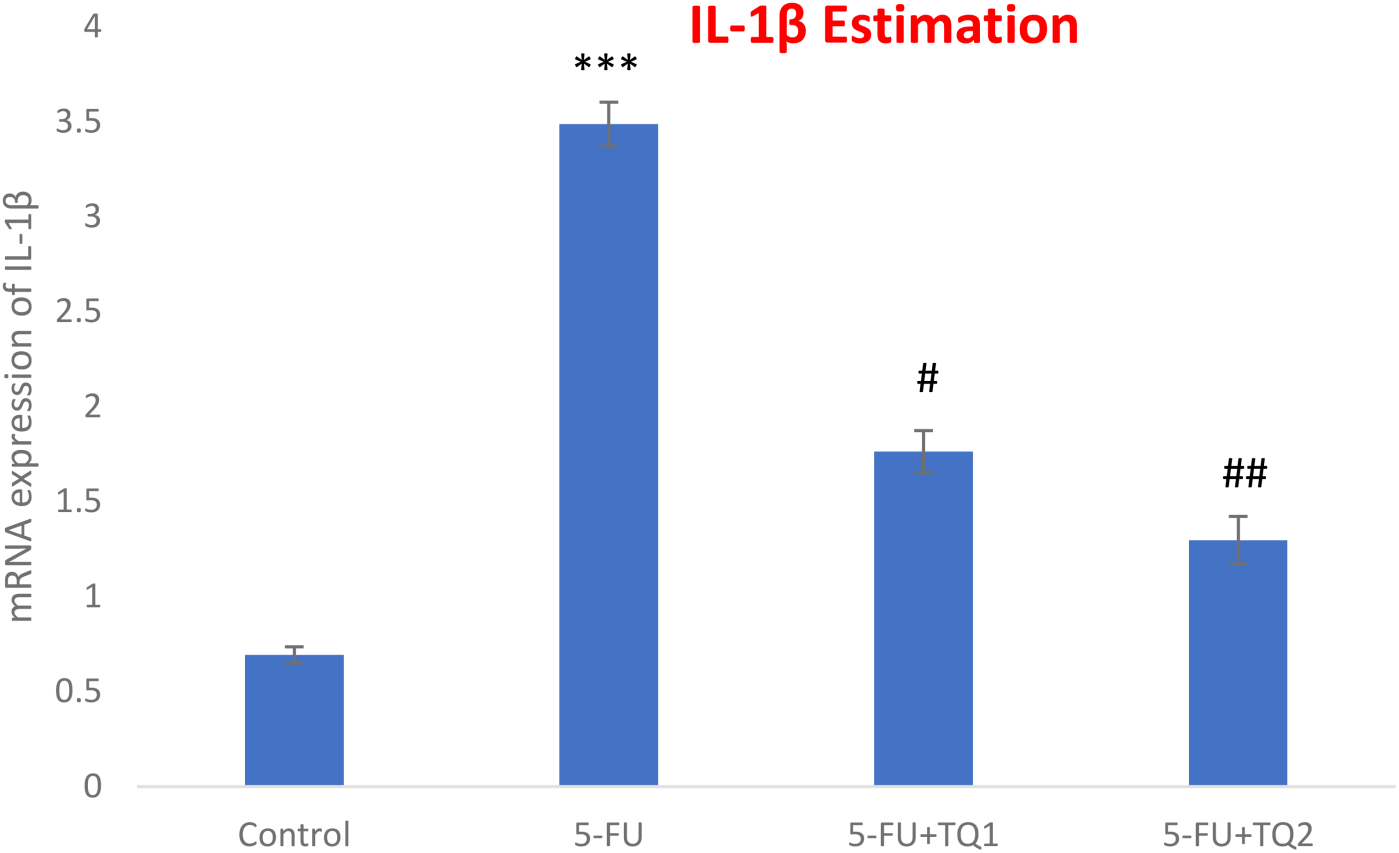
Effect of low and high prophylactic doses of thymoquinone (TQ) on IL-1β in 5-fluorouracil (5-FU)-induced kidney damage. A comparison between the 5-FU-treated group and the control group: ^***^ *p* < 0.001, ^**^ *p* < 0.01, and ^*^ *p* < 0.05. Treatment groups were compared with the 5-FU-treated group: ^#^ *p* < 0.05, ^##^ *p* < 0.01, and ^###^ *p* < 0.001. Group I: normal control, group II: 5-FU-treated (150 mg/kg b.w.), group III: 5-FU-treated (150 mg/kg b.w.) + TQ (lower dose) (50 mg/kg b.w.), and group IV: 5-FU-treated (150 mg/kg b.w.) + TQ (100 mg/kg b.w.).

mRNA expression was assessed to discover the primary mechanism behind the antioxidant efficacy of TQ. Nrf2, HO-1, and IL-1β were checked. 5-FU administration downregulated renal Nrf2 and HO-1 significantly and upregulated IL-1β, indicating inflammation (**Figures 8**–**10**). Also, 5-FU administration upregulated IL-1β, and TQ treatment reversed the effect of these proteins. These data demonstrate the TQ-induced Nrf2 signaling by upregulation of Nrf2 and HO-1 and downregulation of IL-1β, diminishing inflammation and oxidative environment.

### 3.7 TQ affects immunohistochemical expression of p-p38 MAPK and NF-κB

**Figure 11:**
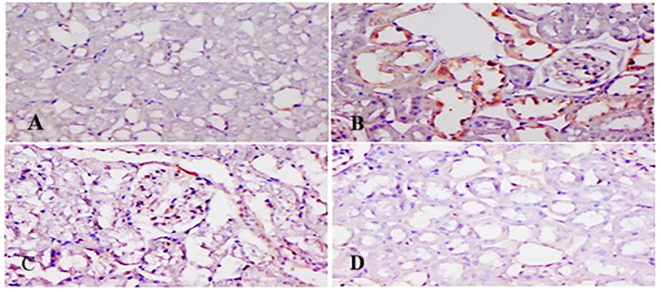
Effect of low and high prophylactic doses of thymoquinone (TQ) on NF-kB in 5-fluorouracil (5-FU)-induced kidney damage. Group I: normal control, group II: 5-FU-treated (150 mg/kg b.w.), group III: 5-FU-treated (150 mg/kg b.w.) + TQ (lower dose) (50 mg/kg b.w.), and group IV: 5-FU-treated (150 mg/kg b.w.) + TQ (100 mg/kg b.w.).

**Figure 12:**
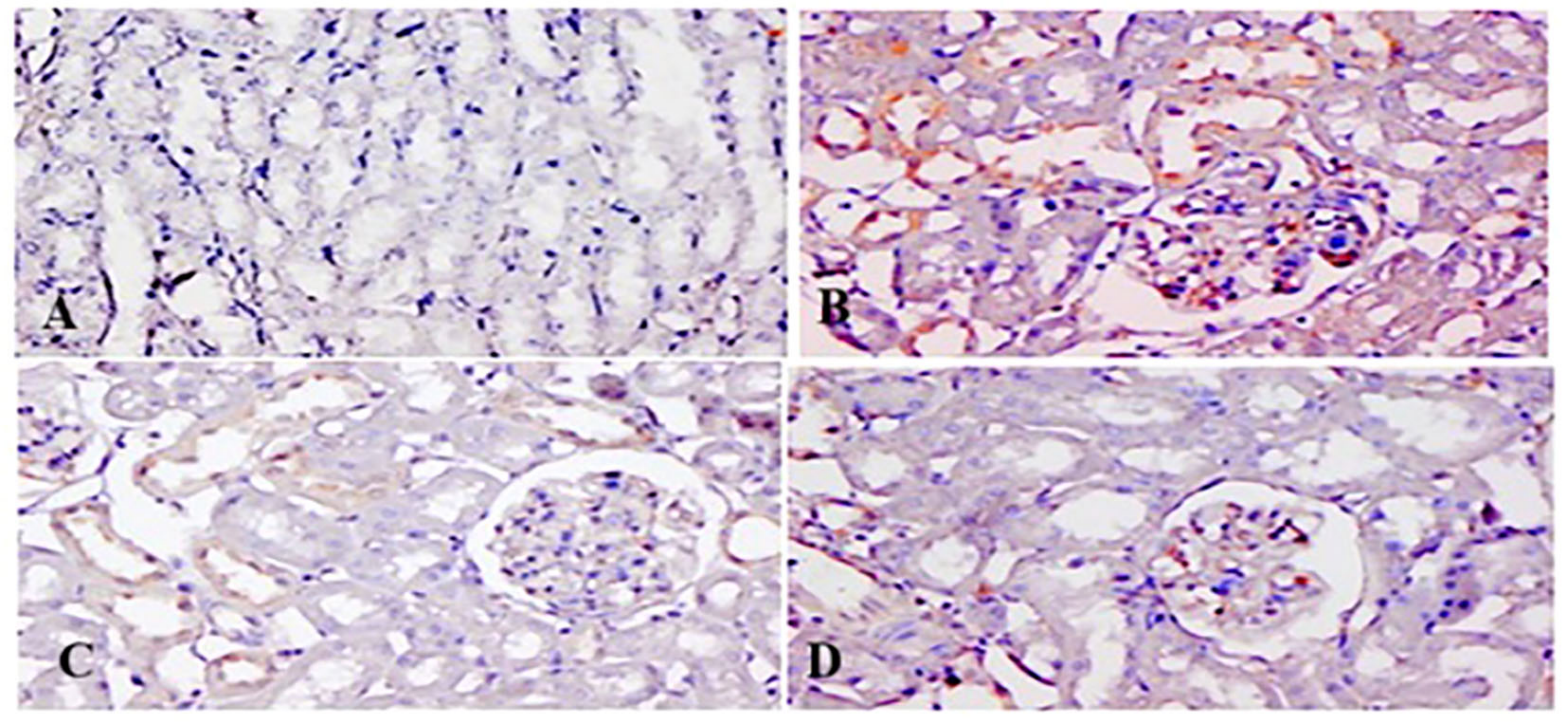
Effect of low and high prophylactic doses of thymoquinone (TQ) on p-p38MAPK in 5-fluorouracil (5-FU)-induced kidney damage. Group I: normal control, group II: 5-FU-treated (150 mg/kg b.w.), group III: 5-FU-treated (150 mg/kg b.w.) + TQ (lower dose) (50 mg/kg b.w.), and group IV: 5-FU-treated (150 mg/kg b.w.) + TQ (100 mg/kg b.w.).

**Figures 11**, **12** show p-p38 MAPK and NF-κB expression in different groups. P38 MAPK and the NF-kB pathway play an imperative part in the progress of 5-FU-initiated kidney toxicity. Intense staining of brown color indicates increased p-p38 MAPK expression in group II than in group I. TQ alleviated brown staining, which indicates less expression of p-p38 MAPK at both, respectively (**Figure 12**). Similarly, there is decreased NF-κB in the control compared to group II, as demonstrated by lesser staining. For immunostaining, NF-κB is indicated by a brown color and hematoxylin stain in a light blue color. TQ treatment decreases the expression of NF-κB in both the groups at both doses, respectively, resulting in attenuation of renal damage (**Figure 11**).

### 3.8 Effect of 5-FU and TQ on histology of kidneys

**Figure 13:**
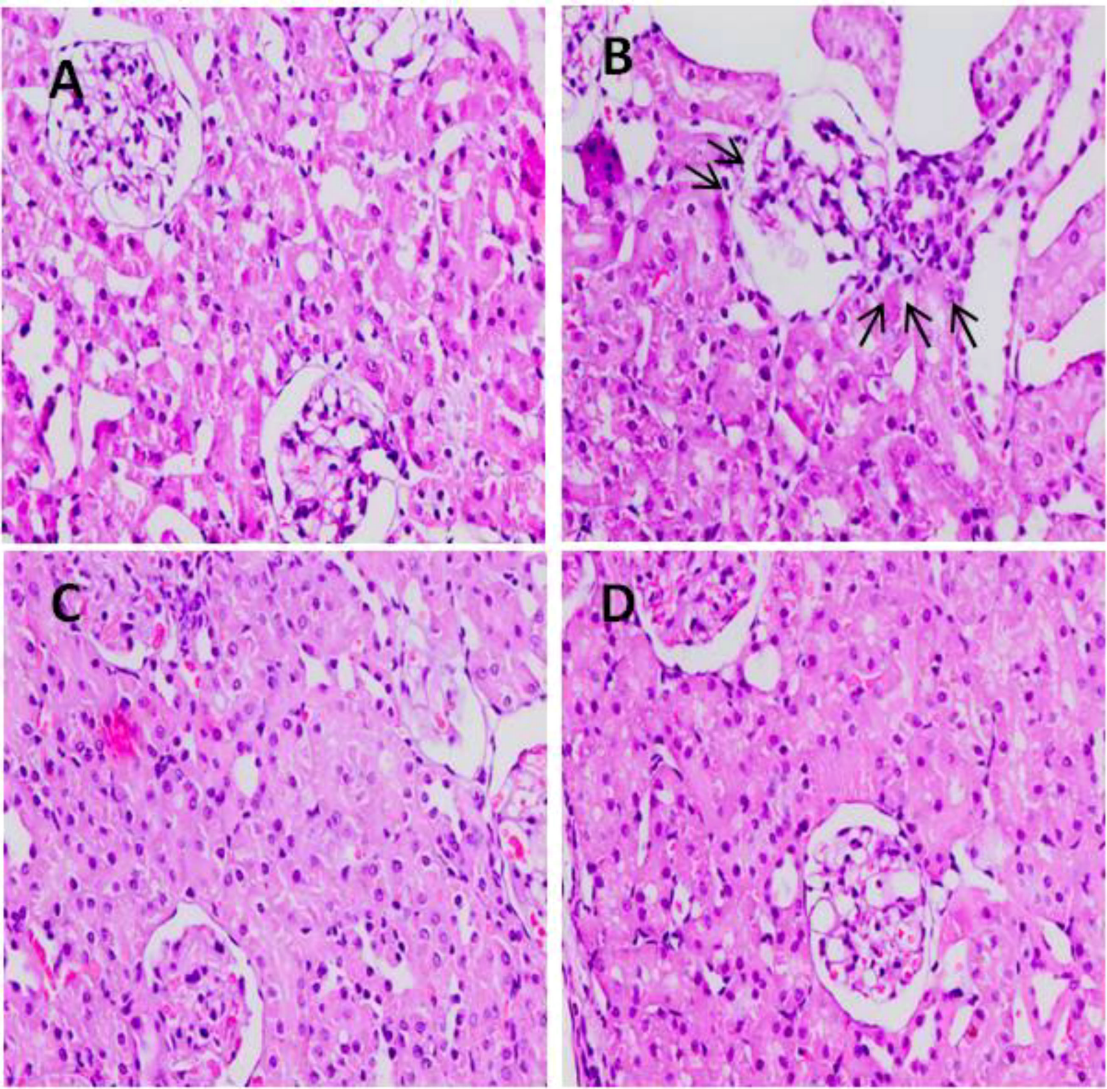
Effect of low and high prophylactic doses of TQ on histology in 5-FU induced kidney damage. Group I: normal control, group II: 5-FU-treated (150 mg/kg b.w.), group III: 5-FU-treated (150 mg/kg b.w.) + TQ (lower dose) (50 mg/kg b.w.), and group IV: 5-FU-treated (150 mg/kg b.w.) + TQ (100 mg/kg b.w.).

Light microscopic examination revealed a standard histological assembly of the renal tissue of the negative control group (**Figure 13A**). However, 5-FU-treated renal tissue showed severe histopathological modifications. Renal corpuscles reveal expanded Bowman’s capsules, congested glomerular capillaries, hemorrhage in interstitial tissue, and infiltration of inflammatory cells in addition to enlarged and congested blood vessels (**Figure 13D**). This demonstrates that pre-, co-, and posttreatment of TQ for 21 days through 5-FU administration intensely reversed the pathological deviation induced by 5-FU in the tissue of the treated rats, evident from vibrant symbols of retrieval as their Bowman’s capsules and glomeruli appeared nearly usual and standard in groups II and IV (C, D).

## 4 Discussion

Kidney injury poses health issues globally, leading to increased morbidity, mortality, and healthcare costs. Moreover, nephrotoxicity caused by chemotherapeutic drugs is a major concern affecting multiple organs like kidneys, followed by acute and chronic kidney injury and renal dysfunction. This kidney dysfunction ascends due to the failure of the kidneys to detoxify and excrete wastes properly. Nearly 20% of kidney injury cases are caused by drugs, which is further increased in the elderly to 66%. 5-FU is a pyrimidine antimetabolite that has serious adverse reactions in the form of kidney damage (15, 23). The existing results interestingly determine the beneficial effects of TQ markedly that were mediated via ROS scavenging, clampdown of MAPKs, and NF-κB in congruence with augmenting Nrf2/HO-1 pathways for the first time.

Free radicals and ROS overproduction are possible mechanisms of pathology by 5-FU-linked renal injury. 5-FU administration contributes to oxidative stress, leading to peroxidation, oxidation, and cross-linking of membrane lipids, proteins, and cellular thiols, leading to the formation of malondialdehyde, ROS, hydroxyl radicals, superoxide anions, and so forth. Furthermore, 5-FU damage elevated renal malondialdehyde, ROS, and H_2_O_2_, as well as alleviated renal antioxidant armory in the present study (38, 39). TQ treatment in groups III and IV reduced MDA, H_2_O_2_, and ROS levels compared to group II, which received only 5-FU. This highlights the antioxidant-boosting potential of TQ and its capacity to diminish oxidative stress (40). Moreover, the GSH cycle is an essential intracellular antioxidant system that sustains cellular structure, function, and survival, and regulates other signaling pathways. ROS and other free radicals generated by 5-FU administration led to a reduction in GSH and an increase in ROS levels (15). Our results show a significant decrease in GSH levels in the 5-FU group. Conversely, TQ treatment markedly restored kidney GSH levels in groups III and IV. Additionally, CAT, GR, GPx, and H_2_O_2_ are key components of the enzymatic antioxidant defense system that protect tissues from oxidative stress and damage. 5-FU weakened the activities of antioxidant defense enzymes due to excessive production of ROS and other free radical formation, as reported (23). The diminished antioxidants and GSH lead to necrosis and further weaken the role/function of kidneys (1, 3). The present results support earlier findings that deciphered that 5-FU administration exhibited a striking decline in the above-said enzymes (15, 40–42). By contrast, concomitant administration of TQ in groups III and IV in our present study increased the activities of CAT, GR, and GPx in comparison to rats that were administered only 5-FU. Consequently, inhibiting/conquering ROS production and augmentation of cellular antioxidant machinery alleviated 5-FU-associated kidney damage (42, 43). This antioxidant machinery shield against the toxic effects of ROS is in agreement with the reports of the improvement of kidney antioxidants in diclofenac, doxorubicin, and acrylamide-associated kidney toxicity studies (36, 44–46). This result supports the suggestion that an upsurge of antioxidants endogenously by TQ to ameliorate oxidative stress in kidneys might be a good approach. Hence, we assume that the renal protective effectiveness of TQ could be ascribed to its antioxidant activity.

Kidney diagnostic serum biomarkers such as Cr, BUN, LDH, and KIM-1 were measured. The kidneys excrete BUN and Cr as waste products of protein metabolism, and an upsurge in these metabolites indicates kidney dysfunction due to impaired function of the glomeruli and tubules, leading to renal necrosis and inflammation. In this context, our results demonstrate that 5-FU induced a substantial rise in the serum biomarkers of renal injury, including BUN, Cr, LDH, and KIM-1, consistent with the findings of Gelen et al. (4, 40). Treatment with TQ in groups III and IV significantly reduced serum levels of BUN, Cr, LDH, and KIM-1 levels, which can likely be attributed to the protective effect of TQ. These findings suggest a revival of glomerular and tubular function, resulting in attenuation of nephrotoxicity. Thus, the current findings clearly demonstrate that TQ exerted renoprotective efficacy, as revealed by the normalization of injury biomarkers. This is in agreement with previous reports indicating attenuation of cisplatin-, lead-, cadmium-, and sodium nitrite-induced renal toxicity through TQ administration (22, 36, 47, 48). Our results showed that TQ reduced ROS generation and lipid peroxidation, enhanced antioxidant levels, and restored serum diagnostic markers in 5-FU-administered rats. Therefore, TQ barred 5-FU-induced kidney damage by inhibiting oxidative injury and reinstating antioxidant defenses and physiological function.

The boosted efficiency of the antioxidant armory could be directly coupled with positive regulation of the Nrf2/HO-1 signaling pathway by TQ treatment. The present results showed the downregulation of Nrf2 and HO-1 abundantly by 5-FU administration, whereas repeated prophylactic TQ treatment potentially caused the reverse effect on the above-mentioned proteins in the present study. Studies provide evidence that Nrf2 controls basal and inducible expressions of numerous cytoprotective and antioxidant genes, reversing oxidative bursts. Although restrained oxidative stress exposure facilitates Nrf2 activation, extreme and continued oxidative stress can weaken Nrf2 signaling in rat kidneys administered with 5-FU (48, 49). Accordingly, weakened Nrf2/HO-1 signaling is directly proportional to sustained ROS generation instigated by 5-FU administration. Renal Nrf2 was upregulated in the TQ-treated groups, leading to the activation of HO-1, GSH, GR, GPx, and CAT in 5-FU-administered rats. Thus, TQ treatment enhanced the antioxidant defense system and reduced ROS production and oxidative damage via the upregulation of Nrf2 signaling. Therefore, Nrf2-boosting drugs/compounds could be beneficial in strengthening antioxidant defenses in kidney injury-related diseases.

Furthermore, activated Nrf2 cross-talks with NF-κB/p38 MAPK signaling and mediates inflammation. NF-κB and its downstream inflammatory cascade inhibition like TNF-α, TGF-β, and IL-1β, have been found to be controlled by Nrf2 and HO-1, thereby regulating the inflammatory process as well. Hence, the antioxidant enzyme induction and inhibition of the NF-kB/p38 MAPK axis by TQ could be elucidated partially by the efficient stimulation of the Nrf2/HO-1 signaling pathway in 5-FU-induced rat kidneys. The charisma of Nrf2 in mediating the inflammation cascade in 5-FU- and TQ-cotreated rats is supported by previous studies demonstrating amplified Nrf2 expression and repressed inflammation in certain preclinical disease models (50, 51). Although little is known regarding NF-κB, TGF-β, and Nrf2 and their contributions to 5-FU-induced nephrotoxicity, they are leading molecules of redox biology and inflammation. Reports show a link between renal pathologies through a considerable rise in NF-κB and TGF-β with subsequent activation of proinflammatory cytokines aggravating tissue inflammation and initiating injury (52, 53).

NF-kB activation and MAPK family proteins are crucial in the regulation of cell differentiation, proliferation, cell death, and inflammatory process mediation. Upon inflammatory stimulation, MAPK P38 kinases crucially promote inflammation in 5-FU-administered rats. Interestingly, rats treated with TQ had a reduction in proinflammatory mediators along with NF-κB/MAPK signaling cascades, thereby unraveling very high-level resistance in 5-FU-induced renal damage and deciphering effective anti-inflammation response. MAPK provokes NF-κB, facilitating activation of downstream genes, which further regulates the proinflammatory retorts that could control the pathological state. Finally, excessive ROS production, lipid peroxidation, and protein adducts upregulated cytokine formation, and the MAPK signaling cascade stimulated NF-κB and its downstream molecules, followed by inflammation and apoptosis leading to renal injury (54, 55). The MAPK superfamily comprises p38 MAP kinases, ERK, and JNK and is an upstream element in the inflammatory pathway. TNF-α-, IL-6-, IL-1β-, and TGF-β-like inflammatory mediators, along with redox imbalance, instigate p38 MAPK and ERK, JNK. MAPK activation has been actively involved in facilitating the stressful measures of 5-FU chemotherapy in animal kidneys, with further JNK and p38 MAPK activation resulting in proinflammatory cytokine formation and, finally, kidney cell death. It could be postulated as a possible mechanism in human kidney dysfunction. Additionally, ERK stimulus activates downstream transcription factors like NF-κB and TNF-α, as reported previously, and similar findings are reported here as well (55, 56). We show that cotreatment with 5-FU and TQ significantly reduced the levels of p-p38MAPK, p-ERK1/2, p-JNK, NF-κB, TNF-α, IL-6, IL-1β, and TGF-β in the treatment groups, indicating that TQ downregulates the p-p-38MAPK/NF-κB signaling pathway involved in 5-FU-induced nephrotoxicity.

Histological variations provide supplementary validation of the kidney injury caused by 5-FU. Our histology data revealed glomerular atrophy, tubular crowding and distention, interstitial hemorrhage, vascular congestion, the presence of proteinaceous material in renal tubules, inflammatory cell infiltration, and other pathological changes in the 5-FU-treated group compared to group 1. These findings align with previous reports. Notably, treatment with TQ prevented 5-FU-induced renal damage, as evidenced by the restoration of structural integrity toward normal healthy kidney architecture in groups III and IV. This histological improvement was consistent with reduced serum levels of Cr, BUN, and KIM-1, indicating a reversal of kidney dysfunction. Similar renoprotective effects of TQ have been reported in cisplatin-, gentamicin-, LPS-, and glycerol-induced nephrotoxicity models in rats (56–59).

## 5 Conclusions

Our results demonstrate that TQ protects against 5-FU-associated renal injury by inhibiting ROS generation and oxidative stress. In addition, TQ mitigated inflammation and restored physiological, structural, and biochemical functions. Activation of the Nrf2/HO-1 signaling pathway, enhancement of antioxidant defenses, regulation of serum markers, and suppression of the NF-kB/p38MAPK pathway—and the resulting reduction in inflammation—appear to be the primary mechanisms underlying TQ’s renal protective effects. Therefore, our findings provide new insights into the protective potential of TQ against 5-FU-induced kidney injury, supporting its possible role as an adjuvant therapy, pending further preclinical and clinical investigations, to improve the quality of life in chemotherapy patients.

## Data Availability

The raw data supporting the conclusions of this article will be made available by the authors, without undue reservation.
